# Collagen production and niche engineering: A novel strategy for cancer cells to survive acidosis in DCIS and evolve

**DOI:** 10.1111/eva.13075

**Published:** 2020-11-04

**Authors:** Mehdi Damaghi, Hidetoshi Mori, Samantha Byrne, Liping Xu, Tingan Chen, Joseph Johnson, Nathan D. Gallant, Andriy Marusyk, Alexander D. Borowsky, Robert J. Gillies

**Affiliations:** ^1^ Department of Cancer Physiology Moffitt Cancer Center and Research Institute Tampa FL USA; ^2^ Department of Oncologic Sciences Morsani College of Medicine University of South Florida Tampa FL USA; ^3^ Center for Immunology and Infectious Diseases Comprehensive Cancer Center Department of Pathology and Laboratory Medicine School of Medicine University of California, Davis Sacramento CA USA; ^4^ Analytic Microscopy Core Moffitt Cancer Center and Research Institute Tampa FL USA; ^5^ Department of Mechanical Engineering University of South Florida Tampa FL USA

**Keywords:** anoikis, cancer evolution, collagen production, extracellular matrix remodeling enzymes, K‐RAS, Niche construction and engineering, SMAD, tumor microenvironment

## Abstract

Growing tumors are dynamic and nonlinear ecosystems, wherein cancer cells adapt to their local microenvironment, and these adaptations further modify the environment, inducing more changes. From nascent intraductal neoplasms to disseminated metastatic disease, several levels of evolutionary adaptations and selections occur. Here, we focus on one example of such an adaptation mechanism, namely, “niche construction” promoted by adaptation to acidosis, which is a metabolic adaptation to the early harsh environment in intraductal neoplasms. The avascular characteristics of ductal carcinoma in situ (DCIS) make the periluminal volume profoundly acidic, and cancer cells must adapt to this to survive. Based on discovery proteomics, we hypothesized that a component of acid adaptation involves production of collagen by pre‐cancer cells that remodels the extracellular matrix (ECM) and stabilizes cells under acid stress. The proteomic data were surprising as collagen production and deposition are commonly believed to be the responsibility of mesenchymally derived fibroblasts, and not cells of epithelial origin. Subsequent experiments in 3D culture, spinning disk and second harmonic generation microscopy of DCIS lesions in patients’ samples are concordant. Collagen production assay by acid‐adapted cells in vitro demonstrated that the mechanism of induction involves the RAS and SMAD pathways. Secretome analyses show upregulation of ECM remodeling enzymes such as TGM2 and LOXL2 that are collagen crosslinkers. These data strongly indicate that acidosis in incipient cancers induces collagen production by cancer cells and support the hypothesis that this adaptation initiates a tumor‐permissive microenvironment promoting survival and growth of nascent cancers.

## 1 INTRODUCTION

The mechanisms underlying the earliest stages of carcinogenesis are not known with certainty. Intraductal proliferation in the breast is common and results from both cell intrinsic alterations common exogenous stimuli such as endocrines and inflammatory factors (Allen & Jones, 2015; Bhatelia, Singh, & Singh, 2014; Damaghi & Gillies, 2016a; Damaghi et al., 2015; Jiang & Shapiro, 2014). In some areas, cells growing into the ductal lumen detach from basement membrane (BM) and either die off (anoikis) or survive within this new microenvironmental niche (Radisky, Muschler, & Bissell, 2002; Weaver et al., 2002). In general, the attachment of epithelial cells to the basement membrane substratum is necessary for their survival, which is promoted by collagen activation of focal adhesion kinases, FAKs (Provenzano, Inman, Eliceiri, & Keely, 2009; Provenzano & Keely, 2009). Substrate detachment is a prerequisite for cell adaptations new microenvironment and anoikis resistance in the breast ductal lumens (Daniela da Silva et al., 2018; Gillies, Robey, & Gatenby, 2008). Since the blood supply for these cells resides in the surrounding stroma, as anoikis‐resistant cells grow within the lumen, this places them away from the source of oxygen so that they become increasingly hypoxic (Gatenby & Gillies, 2004). This hypoxia, along with nutrient deprivation, selects for cells with the phenotype of aerobic glycolysis, also known as the Warburg Effect, WE (Gillies & Gatenby, 2007; Gillies et al., 2008). A significant sequela of aerobic glycolysis is the overproduction of lactic acid (Caruso et al., 2017; Gatenby & Gillies, 2004, 2007; Marchiq & Pouyssegur, 2016). Because intraductal neoplasias are avascular, this results in significant acidification of the extracellular microenvironment, accompanied by upregulation of transporters to export intracellular acids to the extracellular compartment (Counillon, Bouret, Marchiq, & Pouyssegur, 1863; Damaghi, Wojtkowiak, & Gillies, 2013; Gatenby & Gillies, 2008; Parks, Chiche, & Pouyssegur, 2013). Acidosis, in normal cells, induces apoptotic cell death (Park, Lyons, Ohtsubo, & Song, 1999). In carcinogenesis, however, cancer cells must eventually adapt to this harsh acidic environment (Damaghi et al., 2015) leading to phenotypic resistance to acid‐induced apoptosis (Damaghi et al., 2015; Mark Robertson‐Tessi, Gatenby, & Anderson, 2016).

Tumor microenvironment structure predominantly exposed of extracellular matrix (ECM). Although ECM is primarily composed of water, proteins, and polysaccharides, it shows exquisite tissue specificity as a result of its unique composition and topography. Tumor ECM now well understood to be different than normal adjacent ECM (Brownfield et al., 2013). This unique structure of ECM is generated through a dynamic biochemical and biophysical interplay between the various cells in each tissue and their evolving microenvironment (Levental et al., 2009). Collagens are major proteins of the ECM. The unique mechanical properties of collagens are mainly controlled by their structure and density that can affect tumor growth, migration, and metastasis (Morris et al., 2016). Increased deposition and re‐organization of collagens, fibronectin, and proteoglycans is observed in the transition from ductal carcinoma in situ, DCIS, to locally invasive disease and also in later stages and metastasis (Fu et al., 2014; Rybarczyk & Simpson‐Haidaris, 2000). It is commonly assumed that collagen in the ECM is derived from stroma cells such as fibroblasts and macrophages. Collagen surrounding normal ducts has a well‐organized concentric structure. Tumor‐associated collagens are more heterogeneous, either being radially oriented toward vasculature or very unstructured, especially in hypoxic areas (Kakkad et al., 2016). Recently, it has been shown that remodeled stiff collagens are used as “invasion highways” by glioma cells and breast cancer cells which migrate along the collagen fibers aligned toward the blood vessels (Carey et al., 2016; Rahman et al., 2016). ECM collagens can also be degraded by enzymes such as metalloproteinases (Van Doren, 2015) or remodeled through proteinase‐mediated processes by crosslinking enzymes, such as lysyl oxidase, LOX (Salvador et al., 2017) or transglutaminase, TGM2 (Lee et al., 2015; Spurlin, Bhadriraju, Chung, Tona, & Plant, 2009).

Breast cancers are often fast growing in an ever‐changing microenvironment and need to be studied under the light of evolution. Evolutionary dynamics in cancers are driven by the surrounding ecological microenvironment, or niche. The relationship between cancer cells and their niche is reciprocal: adaptation to microenvironment is necessary for cancer cells to survive and thrive within certain niches and in the process of adaptation, cancer cells change their metabolism which affects the composition of the niche(Laland, Odling‐Smee, and Feldman (1999)). Hyperplasia and anoikis‐resistant related acidosis, described above, is an example of such processes and might be referred to as “niche construction.” The consequences of environment modification by cancer cells can affect both their own and other cancer cell's evolution as well as adjacent normal cells and other component of stroma by modifying sources of natural selection.

Niche construction studies on nascent cancer evolution are scarce. In this work, we address the role of niche construction that occurs during the early stages of breast cancer. The breast cancer ecosystem consists of cancer cells and stroma including the extracellular matrix (e.g., collagen, fibronectin, proteoglycans), immune system components, and vasculature. Of these, the primary structural component of the tumor niche is ECM. It is well known that ECM plays a key role in development, survival, morphogenesis, and function of cells in mammary glands (Fata, Werb, & Bissell, 2004; Katz & Streuli, 2007).

Although the concept of niche construction is under‐developed, there has been some recent work on niche construction theory recognizing environmental modification by organisms and its consequences as “ecological inheritance” to be evolutionary processes (Odling‐Smee, Erwin, Palkovacs, Feldman, & Laland, 2013). Two aspects of niche construction are distinguished as (a) environment alteration and (b) subsequent evolution in response to constructed environments (Odling‐Smee et al., (2013)). There are numerous examples in nature of large organisms in nature engineering their habitats or constructing niche, leading to an evolutionary response in their species such as orb‐web, ants, bees, wasps, and termites who construct their nests and habitats that are themselves the source of selection of their own species (Firn & Jones, 2000; Jones, Lawton, & Shachak, 1997; Lawton & Jones, 1993).

In this work, we identify an important component of niche construction in early stage breast cancers: the acid‐induced production of collagens by nascent pre‐cancerous and locally invasive cancer cells. Herein, we show that acid‐adapted cancer cells switch to a phenotype and that is resistant to anoikis and is more adhesive to matrix proteins such as collagen. We then identify by discovery proteomics that adaptation of pre‐malignant cancer cells to chronic acidosis involves upregulation of collagen producing (PLODs) and remodeling (LOXs and TGM2) enzymes which are required for collagen export from cells and which is a precursor to crosslinking with lysyl oxidase. Second harmonic generation (SHG) microscopy shows significant collagen deposition within the lumens of DCIS, which are devoid of stromal cells. Multiple molecular techniques confirm the production of rare collagens by acid‐adapted pre‐malignant and cancerous breast epithelial cells in vitro. Defined molecular mechanisms of this adaptation phenotype include K‐RAS activation and subsequent downstream pathway activation. These discoveries help to unravel the cellular and molecular mechanisms of the initiation and maintenance of a tumor‐promoting microenvironment. This, in turn, may inform new design of combination therapies or niche re‐engineering with potential impact both treatment and prevention.

## 2 METHOD

### 2.1 Cell culture and in vitro acid adaptation

MCF‐7, MCF10‐AT, MDA‐mb‐231, and MCF10 and MCF10AT cells were acquired from American Type Culture Collection (ATCC, Manassas, VA, 2007–2010) and were maintained in DMEM‐F12 (Life Technologies) supplemented with 10% fetal bovine serum (HyClone Laboratories). Growth medium was further supplemented with 25 mmol/L each of PIPES and HEPES and the pH adjusted to 7.4 or 6.7. For MCF10A and MCF10AT, media were further supplemented with 0.5 μg/ml hydrocortisone, 100 ng/ml cholera toxin, 20 ng/ml human epidermal growth factor, and 10 μg/ml insulin.

Cells were tested for mycoplasma contamination and authenticated using short tandem repeat DNA typing according to ATCC’s guidelines. For acute acidosis, cells were exposed to acidic media for 72 hr. To achieve acid adaptation, cells were chronically cultured and passaged directly in pH 6.7 medium for more than 3 months.

### 2.2 SILAC labeling and proteomics

This technique was described in details in our previous publication (Damaghi et al., 2015).

#### 2.2.1 Secretome proteomics

Acid‐adapted and non‐adapted cancer cells were cultured in 10 cm dishes at 50% confluency. After 24 hr, their media were replaced with serum‐free media, which was collected after 12 hr of incubation and lyophilized. Proteins were dissolved in denaturing lysis buffer containing 8 M urea, 20 mM HEPES (pH 8), 1 mM sodium orthovanadate, 2.5 mM sodium pyrophosphate, and 1 mM β‐glycerophosphate. A Bradford assay was carried out to determine the protein concentration. The proteins were reduced with 4.5 mM DTT and alkylated with 10 mM iodoacetamide. Trypsin digestion was carried out at room temperature overnight, and tryptic peptides were then acidified with 1% trifluoroacetic acid (TFA) and desalted with C18 Sep‐Pak cartridges. LC‐MS/MS analysis was carried out with a nanoflow ultra high‐performance liquid chromatograph (RSLC, Dionex, Sunnyvale, CA) coupled to an electrospray mass spectrometer (Q‐Exactive Plus, Thermo, San Jose, CA). The sample was first loaded onto a pre‐column (2 cm × 100 µm ID packed with C18 PepMap100 reversed‐phase resin, 5 µm particle size, 100 Å pore size) and washed for 8 min with aqueous 2% acetonitrile and 0.04% trifluoroacetic acid. The trapped peptides were eluted onto the analytical column, (C18 PepMap100, 75 µm ID × 25 cm, 2 µm particle size, 100 Å pore size, Dionex, Sunnyvale, CA). The gradient was programmed as: 95% solvent A (2% acetonitrile + 0.1% formic acid) for 8 min, solvent B (90% acetonitrile + 0.1% formic acid) from 5% to 38.5% in 60 min, then solvent B from 50% to 90% B in 7 min and held at 90% for 5 min, followed by solvent B from 90% to 5% in 1 min and re‐equilibration for 10 min. The flow rate on analytical column was 300 nl/min. Sixteen tandem mass spectra were collected in a data‐dependent manner following each survey scan using 15‐s exclusion for previously sampled peptide peaks. MS1 resolution was set at 70,000 and MS/MS resolution was set at 17,500 with ion accumulation (maxIT) set to 50 ms. MaxQuant (version 1.2.2.5) was used to identify and quantify the proteins.

### 2.3 qRT‐PCR

qRT‐PCR was done using the iScript Real‐time PCR kit with SYBR Green (Cat# 170–8893 Biorad) on a Biorad System. Primers were derived from Primer Depot (primerdepot.nci.nih.gov/). 100ng of mRNA was used in each well for 20 μL reaction. RNeasy kit from Qiagene was used for all RNA purification with the company instruction. GAPDH or actin was used as an internal control for all experiments with water and no primer controls.

### 2.4 Western blotting

Acid‐adapted and non‐adapted MCF10‐AT, MCF‐7, and MDA‐MB‐231 cells were grown with the same number of passages and used for whole‐protein extraction. Lysates were collected using RIPA buffer containing 1 × protease inhibitor cocktail (P8340; Sigma‐Aldrich). Twenty micrograms of protein per sample was loaded on polyacrylamide–SDS gels, which later were electrophoretically transferred to nitrocellulose. Membranes were incubated with primary antibodies against rabbit polyclonal LAMP2 (1:1,000, ab18529 Abcam), mouse monoclonal PLOD2 (1:1,000, Fischer Scientific), Rabbit polyclonal PLOD1(LS‐C163796‐400), Rabbit monoclonal TGM2 (ab109200 Abcam), K‐Ras, N‐Ras, and H‐Ras (Santa Cruz Biotechnology) and GAPDH (1:4,000, antirabbit; Santa Cruz Biotechnology).

### 2.5 Immunofluorescence

Cells cultured at pH 6.7 chronically and pH 7.4 of with the same number of passages were rinsed with PBS, fixed in cold 4% paraformaldehyde for half an hour, and then blocked with 4% bovine serum albumin in PBS. Samples were incubated with LAMP2 rabbit polyclonal primary antibody (1:100; ab 37024 Abcam) and secondary Alexa‐Fluor 488 antirabbit (1:500) antibody. Coverslips were mounted using ProLong Gold Antifade Reagent (Life Technologies) and images were captured with a Leica TCS SP5 (Leica) confocal microscope.

#### 2.5.1 Spinning disk cell adhesion measurement

To measure the strength of cellular adhesion, a hydrodynamic flow chamber is filled with a fluid of known viscosity and density (Elineni & Gallant, 2011; Gallant, Michael, & Garcia, 2005). Inserted into the chamber is a spinning shaft that holds via vacuum a glass coverslip on which cells have adhered. The cells are therefore exposed to the shear force of the laminar flow at a given rotational speed. The shear stress varies linearly with radius:

$$\tau =0.8r\sqrt{\rho \pi \omega^{3}}$$

where *r* is the radial position along the substrate, *μ* is the viscosity, *ρ* is the density of the solution, and *ω* is the angular velocity. After spinning for 5 min, the remaining adherent cells were fixed in 3.7% formaldehyde, permeabilized with 0.1% Triton X‐100, and stained with Hoechst dye to identify the nucleus. The number of adherent cells was counted at specific radial positions using an Eclipse Ti‐U fluorescent microscope (Nikon Instruments, Melville, NY) fitted with a motorized stage and NIS‐Elements Advanced Research software (Nikon Instruments). Sixty‐one fields were analyzed per substrate and the number of cells at specific radial locations was then normalized to the number of cells at the center of the substrate where negligible shear stress was applied to calculate the fraction of adherent cells *f*. The detachment profile (*f* vs. *τ*) was then fit with a sigmoid curve

$$f=1/\left(1+e^{\left[b\left(\tau ‐\tau 50\right)\right]}\right)$$

The shear stress for 50% detachment (*τ*_50_) was used as the mean cell adhesion strength.

#### 2.5.2 Spheroid assay

Perfecta3^®^96‐Well Hanging Drop Plates and low attachment U shape plates were used to grow the primary spheres containing 10,000 cells (acid‐adapted MCF‐7 or non‐adapted MCF‐7) without using any matrix such as Matrigel or collagen. After 24 hr, the spheres media were changed to acid or normal media. Acid‐adapted MCF7 cells grow robust spheres around day7 while NA MCF7 never grow real sphere. Incucyte microscope was used to image the sphere growth. Data were analyzed using custom‐built Incucyte software.

#### 2.5.3 Soft agar assay

Soft agar colony formation assay is used to study the anchorage–independent growth of tumor cells. 1 million NA and AA MCF7 cells are trypsinized, counted, and dissolved in 1 ml DMEM with 10% FBS. 4% agar was melted by microwave and kept in 56 C water bath to be used as bottom layer. To prepare the bottom layer, 4:1 ratio of 4% agar:DMEM media with 10% serum was mixed and poured in 6‐well plates (1 ml per well). The plates were placed in hood to let the gel solidify. For top layer, 0.4% agar gel was made by adding the DMEM medium with 10% serum. 100 cells were added per milliliter of the mixture and added on top of the bottom layer. Plates were placed in 37C incubator for 2–3 weeks, and colonies were counted by microscopy or crystal violet staining.

### 2.6 Active Ras measurement

Ras superfamily of small GTPases is active when bound to GTP and inactive when the triphosphate is hydrolyzed to GDP. The Active GTPase pull‐down and Detection Kits enrich Ras active form using a GST‐protein binding‐domain fusion that is selective for active Ras. The amount of enriched active Ras is measured using specific antibody and Western blotting. We used Active Ras Detection Kit #8821 from cell signaling technology that uses GST‐Raf1‐RBD fusion protein to bind the activated form of GTP‐bound Ras, which can then be immunoprecipitated with glutathione resin. Ras activation levels are then determined in western using a Ras mouse monoclonal antibody.

### 2.7 Animal experiment

All animals were maintained in accordance with IACUC standards of care in pathogen‐free rooms, in the Moffitt Cancer Center and Research Institute (Tampa, FL) Vivarium. One week before inoculation with tumor cells in the mammary fat pads, female nu/nu mice 6–8 weeks old (Charles River Laboratories) were placed in two cohorts per experiment randomly selected. Acid‐adapted and non‐adapted MCF7 and MCF10AT cells were mixed with Matrigel in cold PBS and were injected into mammary fat pad of animals. The tumor size was monitored by caliper three times a week and by ultrasound every week. When the tumor reached 1,500 mm^3^ of size, animals were humanely killed and tumors were extracted, fixed in 10% formalin, paraffin embedded, and further processed for IHC.

### 2.8 Microarray analysis

Affymetrix expression data for ColXa1 and Col11a1 genes in patient samples were produced from publicly available data sets of Moffitt Cancer Center patients. The CEL files for the tumor samples were downloaded from the Gene Expression Omnibus (GEO) database (http://www.ncbi.nlm.nih.gov/geo/), data series GSE2109. Normal tissue data were from the GEO data series GSE7307, Human Body Index. The CEL files were processed and analyzed using the MAS 5.0 algorithm (Affymetrix) and screened through a rigorous quality control panel to remove samples with a low percentage of probe sets called present by the MAS 5 algorithm, indicating problems with the amplification process or poor sample quality; high scaling factors, indicating poor transcript abundance during hybridization; and poor 30/50 ratios, indicating RNA degradation either before or during processing. The remaining samples were normalized to the trimmed average of 500 in the MAS 5 algorithm before comparison of the expression values across tumors and normal samples.

### 2.9 Immunohistochemistry

For human tissues, a TMA containing formalin‐fixed and paraffin‐embedded human breast tissue specimens was constructed in Moffitt Cancer Center histology core. The TMA contains 27 normal breast tissues, 30 DCIS, 48 invasive ductal carcinomas without metastasis, 49 invasive ductal carcinomas with metastasis, and 48 lymph node macro‐metastases of breast cancer. Cores were selected from viable tumor regions and did not contain necrosis. A 1:200 dilution of anti‐LAMP2b (#ab18529, Abcam) and 1:200 of anti‐TGM2 (#ab109200, Abcam) was used as primary antibody. Normal placenta was used as a positive control for LAMP2 and normal human kidney for TGM2. For the negative control, an adjacent section of the same tissue was stained without application of primary antibody, and any stain pattern observed was considered as nonspecific binding of the secondary.

Immunohistochemical analysis was conducted using digitally scanning slides. The scoring method used by the pathologist reviewer to determine (a) the degree of positivity scored the positivity of each sample ranged from 0 to 3 and were derived from the product of staining intensity (0–3^+^). A zero score was considered negative, score 1 was weak positive, score 2 was moderate positive, and score 3 was strong positive. (b) The percentage of positive tumors stained (on a scale of 0–3).

#### 2.9.1 Collagen content measurement in cells

As briefly described in following: MCF7 and AA‐MCF7 cells were seeded on a glass bottom plate stained with CNA‐35‐GFP and fixed with 4% paraformaldehyde. The plates were imaged using a confocal microscope equipped with SHG system enabling us to image fluorescent and SHG at the same time. The results showed more collagen and fibrillar structure in both fluorescent imaging of CNA‐35‐GFP and SHG, respectively (Figure 2c).

#### 2.9.2 Statistical analysis

A two‐tailed unpaired Student's *t* test was performed to compare treated and non‐treated groups of animals and also for tumors against normal cells to determine statistical significance. The significance level was set as *p* < .05.

## 3 RESULTS

### 3.1 Acid‐adapted cells are resistant to anoikis

It has been known for more than a decade that center of ducts in glandular cancer models such as breast or prostate cancer is one of the most acidic habitats in the whole tumor ecosystem (Figure S1; Damaghi & Gillies, 2016a; Damaghi et al., 2015; Ibrahim‐Hashim et al., 2017). Mechanisms allowing tumor cells to tolerate acidosis have been studied extensively by our group (Chen et al., 2008; Damaghi et al., 2015; Wojtkowiak et al., 2012) and others (Chen et al., 2008; Koutcher, Fellenz, Vaupel, & Gerweck, 1988). However, the impact of acidosis on tumor niche and its components such as collagens and cancer evolution are less understood. It was proposed that cell death imposed by loss of basement membrane attachment (“anoikis”) is one of the first evolutionary barriers that cancer cells reach in their early progression (Gatenby & Gillies, 2008) but the impact of acidosis on this process was unknown.

**Figure 1:**
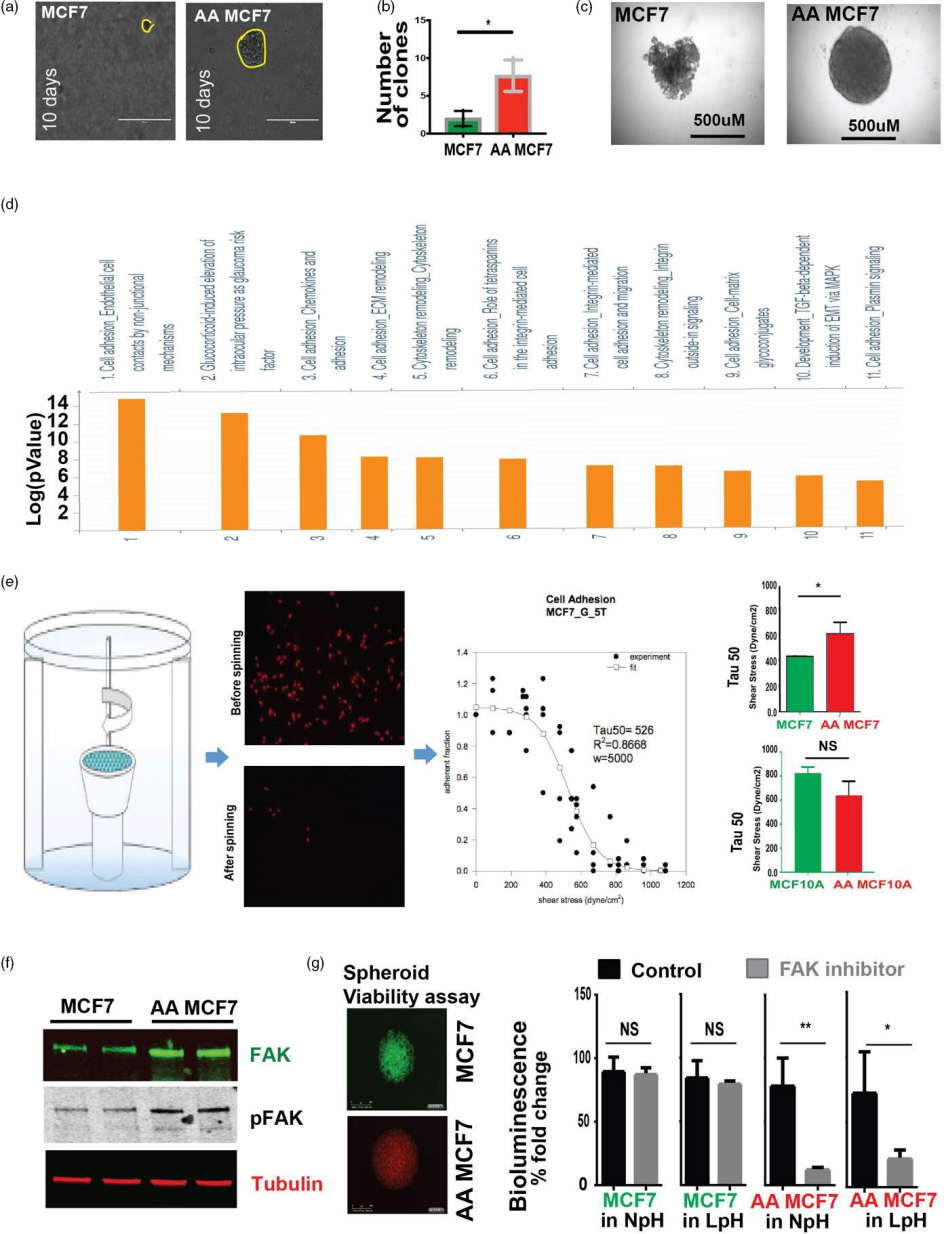
Acid adaptation prevents anoikis as a survival mechanism of early lesion cancer cells. (a) (b) Soft Agar clonogenic assay on MCF7 and AA MCF7 cells. AA MCF7 cells grow more and bigger clones than non‐adapted MCF7. (c) Spherogenicity assay on MCF7 and AA MCF7 cells. Acid‐adapted MCF7 cells grow sphere in ultra‐low adhesion plates contrary to the non‐adapted cells. (d) Proteomics analysis of AA MCF7 versus MCF7 cells. The analysis was applied on filtered proteins that have role in ECM and anoikis. More than 80% of the top ten pathways that were upregulated in acid‐adapted cells belong to cell adhesion, ECM–cell adhesion, and integrin‐mediated cell adhesion pathways implying the role of acid adaption in regulation of those pathways. (e) Spinning disk experiment to measure cell–matrix adhesion. AA MCF7 cells are more adhesive than MCF7 while the normal MCF10A cells have opposite behavior. (f) Western blot of FAK and pFAK on lysates from AA and NA MCF7 cells. (g) 3D viability assay of AA MCF7 and MCF7 spheroid treated with FAK inhibitors

To investigate this phenomenon, we stained 50 DCIS biopsy core from TMA4 of Moffitt tissue core for the acid‐adaptation marker LAMP2b (Damaghi et al., 2015). We observed that plasma membrane‐bound LAMP2b was overexpressed in the periluminal regions of early DCIS breast lesions (Figure S1) prompting us to hypothesize that these acid‐adapted cells are resistant to anoikis. To examine this in vitro, we tested anchorage‐independent survival in soft agar by using MCF7 cells (NA MCF7; non‐adapted control cells) and their acid‐adapted counterparts (AA MCF7) that have been grown in acidic (pH6.5) media for more than 20 passages; until their growth rate at this pH matches unselected cells at physiological pH (pH7.4; Damaghi et al., 2015). The results showed AA MCF7 cells grew significantly more and larger colonies in soft agar (Figure 1a,b) compared to NA MCF7. To corroborate this finding, we examined anchorage‐independent growth of AA and NA MCF7 with a spherogenic assay in an ultra‐low adhesion U shape plate in both pH 7.4 and 6.5. Whereas NA MCF7 cells only formed loose clumps containing relatively fewer cells; AA MCF7 formed large, well‐defined spheres in both low and high pH culture conditions (Figure 1c). The dramatic differences in colony shapes under low attachment conditions suggested that acid adaptation has increased cell adhesion. These two experiments indicated that acid‐adapted cells are not only capable of anchorage‐independent growth but also proliferation which is necessary for their evolution in center of the duct. To study the molecular mechanisms associated with this adaptation strategy, we analyzed previously generated SILAC proteomic data (Damaghi et al., 2015) from NA vs. AA MCF7 cells with GeneGo software; focusing on membrane proteins and ECM components. These analyses showed that cell adhesion, ECM remodeling, and cytoskeleton remodeling proteins were elevated in AA MCF7 cells compared to non‐adapted MCF7 (Figure 1d). Given the elevated expression of ECM components, as well as molecules responsible for cell–matrix adhesion, we then asked whether AA MCF7 cells are capable of stronger adhesion to ECM. We performed adhesion strength experiments on NA MCF7 and AA MCF7 cells as well as NA and AA MCF10A, immortalized mammary epithelial cells, as non‐cancer control. These experiments showed that AA MCF7 cells were more adhesive than NA MCF7 cells, consistent with the proteomic data. In contrast, acid adaptation insignificantly reduced adhesion of MCF10A cells, implying differential matrix adhesion responses of cancer cells to acid adaptation, compared to normal cells (Figure 1e). Different phenotype in these untransformed cells suggests that this phenomenon might selectively was regulated in cancer cells that can be used as vulnerability. Next, we asked whether increased self‐generated adhesion induced by acid adaptation might be responsible for an increased anchorage‐independent survival. First, we examined the activation status of focal adhesion kinase (FAK) to investigate if AA MCF7 has higher levels of FAK activity. Indeed, we found that levels of FAK activity were significantly higher in AA MCF7 cells compared with NA MCF7 cells (Figure 1f). Next, we asked whether an increased FAK activity was contributing to increased anchorage‐independent survival of AA MCF7 cells. The hypothesis was that if cancer cells can produce their matrix that can be used by FAKs the viability of the spheres should be affected by FAK inhibitors. To this end, we compared the impact of FAK inhibition on survival under 3D growth between AA and NA MCF7 cells. Using 3D viability assay from Promega (Cell titer‐Glo 3D), we found that FAK inhibitors (FAK Inhibitor‐14) decreased the viability of AA MCF7 cells both in normal pH and acidic media, whereas NA MCF7 cells were unaffected, suggesting that increased cell adhesion to autonomously produced ECM might be responsible for increased anchorage‐independent survival during acid adaptation under either acidic or normal pH conditions (Figure 1g).

### 3.2 Acid adaptation promotes collagen production of cancer cells

**Figure 2:**
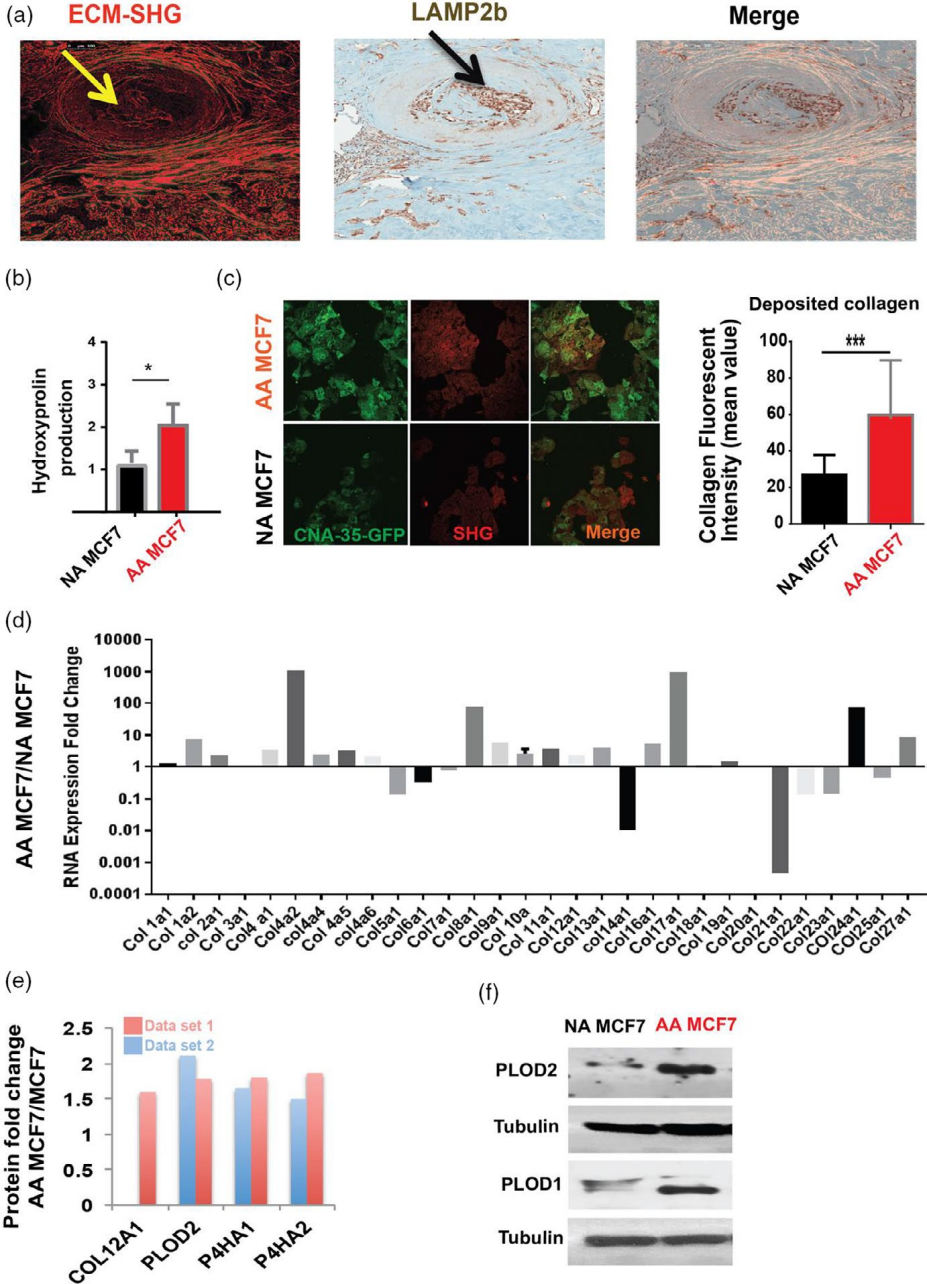
Acid adaptation promotes collagen production of cancer cells. (a) Second harmonic generation (SHG) microscopy of breast tumors confirms the existence of collagen in the center of ducts in DCIS breast tumors. DCIS is the most acidic part of the tumor due to avascular nature of early carcinoma. (b) Hydroxyproline assay showed higher amount of hydroxyproline in acid‐adapted cancer cells compared to non‐adapted ones. Hydroxyproline is an essential precursor of the collagen fibers that is necessary for its stability. The data are repeated in three biological replicates, and data are presented as mean with *SD* as error bar. (c) Fluorescent confocal microscopy coupled with second harmonic generation operator confirmed the higher expression of collagen and fibrillary structures in acid‐adapted cancer cells. Collagen production was measured using SHG and confocal microscopy on paraformaldehyde fixed breast cancer cells. CNA‐35‐GFP marker was used to distinguish collagen from other repetitive structure detected by SHG. Acid‐adapted cells produce more collagen significantly. Data are shown as standard deviation with mean as error bars with three separated biological replicates. (d) Expression pattern of collagen genes in acid‐adapted MCF7 cells against non‐adapted ones. The expression response pattern is quite heterogeneous and varies from over expression to lower expression. The y axis scale is in logarithmic some genes such as Col17a1 are highly expressed in acid‐adapted cells while Col21a1 is overly downregulated. (e) SILAC proteomics analysis of AA MCF7 against NA MCF7 cells revealed increased expression of collagen production enzymes such as PLODs, and P4Has as well as some collagens in acid‐adapted MCF7 cells. Data are presented in two set that are two separate runs of mass spectrum. F) Western blot validation of PLOD higher expression in acid‐adapted cells compared to non‐adapted one

Above, we observed adhesion of cancer cells to matrix as a strategy to become anchorage independent for growth. Within ductal lumens, there are no matrix‐producing fibroblasts. In ductal hyperplasia and carcinoma in situ, the only cells in periluminal volumes are expected to be epithelial‐derived pre‐cancerous and cancerous cells. Therefore, we hypothesized that acid‐adapted cancer cells are either truly matrix independent, or they are producing their own matrix to allow survival. To investigate this, we examined the ECM of breast tumors by imaging Moffitt cancer patients’ breast tumor samples using second harmonic generation (SHG) microscopy. The advantage of SHG imaging is that specific staining is not required and it can be performed on IHC‐stained formalin‐fixed paraffin‐embedded (FFPE) slides to acquire expression data of proteins at the same time of fibrillar structures. We stained whole mount breast tumors samples for the acid‐adaptation marker, LAMP2b, (Damaghi & Gillies, 2016b; Damaghi et al., 2015) and subsequently imaged these same samples with bright‐field microscopy for LAMP2b and SHG for fibrillar structures (Figure 2a). We found fibrillar structure inside the ducts and distribution of SHG signals correlate with the presence of acid‐adapted cells expressing LAMP2b.

To further investigate if the matrix observed by SHG contains collagen and also the effect of acid adaption on collagen production by cancer cells, we measured the hydroxyproline component of conditioned media from NA MCF7 and AA MCF7 cancer cells. Hydroxyproline is a major amino acid in collagen that is generated post‐translationally and has a critical role in collagen thermostability. Because of high specificity of hydroxyproline to collagen, it can be used as an indicator of collagen content in isothermal conditions. Our result showed that the hydroxyproline content in conditioned media was increased in response of acid adaptation in MCF7 (Figure 2b) cells. As cross validation of our finding in vitro, the collagen‐binding protein, CNA‐35‐GFP, (Shi et al., 2014) was then used to measure the collagen content of the cells and collagen deposited from AA MCF7 and NA MCF7 cells grown on glass bottom dishes (Shi et al., 2014). These same samples were also imaged with second harmonic imaging (SHG) which is also sensitive to regularized ECM structures (Figure 2c). These experiments also showed the higher levels of collagens produced by acid‐adapted cancer cells.

To uncover what types of collagens are being induced by acid adaptation, we investigated the expression profile of 30 collagen genes in AA MCF7 and NA MCF7 using qRT‐PCR (Figure 2d). This showed a very heterogeneous response to acid adaptation in terms of collagen production. Some collagens such as Col17a1, Col4a2, Col8a1, Col9a1, Col10a1, Col11a1, and Col24a1 showed increased expression in acid‐adapted cells, whereas Col5a1, Col6a1, Col7a1, Col14a1, and Col21a1 mRNA levels were lower, and a third group including Col3a1, Col18a1, and Col19a1 that showed no or very small change in their expression (Figure 2d). These results implied the different roles of different collagens in response to chronic acidosis and their possible differential role in the development and progression of tumors inside the duct.

mRNA levels do not necessarily predict the levels of expressed proteins, especially in long‐lived structural proteins. To study different types of collagen overexpression in acid‐adapted cells at protein level, we searched our SILAC proteomic data on AA MCF7 versus NA MCF7 cells for matrix proteins and specifically collagen‐related proteins. The proteomic data showed that collagen 12a1, and collagen modifying enzymes, such as PLODs and P4HAs, were increased in acid‐adapted cells (Figure 2e). Notably, col12a1 was also upregulated at the mRNA level. PLOD proteins are membrane‐bound homodimeric enzymes localized to the cisternae of the rough endoplasmic reticulum and catalyze the hydroxylation of lysyl residues in collagen peptides (Qi & Xu, 2018). These hydroxylysyl groups form attachment sites for carbohydrates, as well as substrates for lysyl oxidase catalyzed intermolecular crosslinks. P4HA proteins are components of prolyl 4‐hydroxylase, which catalyzes the formation of 4‐hydroxyproline, which is necessary to the proper three‐dimensional folding and thermostability of newly synthesized procollagen peptides. We validated the higher production of PLOD1 and PLOD2 in acid‐adapted cells with Western blotting (Figure 2f). We also looked at PLOD expression in the Protein Atlas (https://www.proteinatlas.org) and found PLOD in the center of the periluminal area of DCIS (Figure S2), which we have shown is most acidic (Figure 2a). The role of PLODs in some cancers progression has recently been reported (Li, Lian, Huang, Huang, & Xiao, 2020). We conclude collagen production is elevated in acid‐adapted cancer cells.

### 3.3 Acid‐induced collagen production is controlled by SMAD proteins and RAS activity

**Figure 3:**
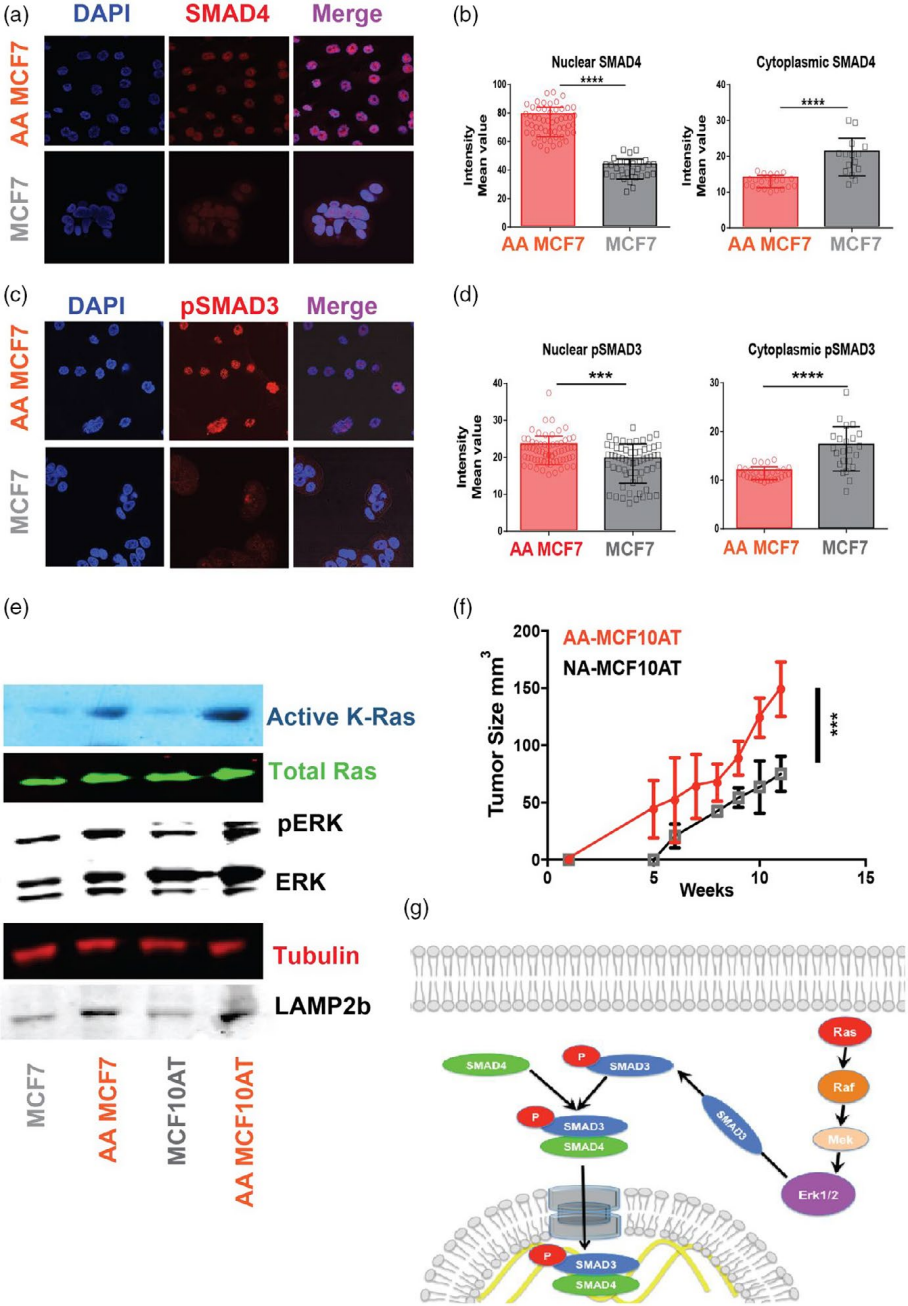
Acid‐induced collagen production is controlled by SMADs and K‐Ras. (a) Immunocytochemistry (ICC) of acid‐adapted MCF7 and non‐adapted MCF7 for SMAD4. SMAD4 is a transcription factor that controls the production of collagen when it is located in nucleus. (b) Nuclear localization of SMAD4 versus its cytoplasmic localization revealed the higher nuclear localization of this protein in acid‐adapted cells. (c) pSMAD3 staining of acid‐adapted and non‐adapted MCF7 cells showed upregulation of pSMAD3 in acid‐adapted cells. (d) Nuclear and cytoplasmic localization analysis of pSMAD3. SMAD3 is a cytoplasmic protein that binds to SMAD4 in its phosphorylated form and translocates SMAD4 into the nucleus. pSMAD3 has higher nuclear expression and lower cytoplasmic expression in acid‐adapted MCF7 cells compared to non‐adapted MCF7. (e) Western blot analysis shows Ras activation and downstream protein regulations. K‐Ras is activated in AA MCF7 and AA MCF10AT cancer cells. Downstream of K‐Ras is ERK that gets activated through phosphorylation. Western blot shows higher p‐ERK in acid‐adapted cells compared to controls. Higher level of LAMP2b is a marker of acid adaptation that shows the cells are completely acid adapted. (f) Tumor growth of acid‐adapted MCF10AT and non‐adapted ones injected to nude mice. AA MCF10AT cell frequency of forming tumor is higher, and the tumors that are formed grow faster. (g) Schematic of Ras activation role in collagen production in cancer cells. We proposed Ras activation phosphorylate ERK that can activate SMAD3 through phosphorylation. pSMAD3 will translocate SMAD4 to nucleus to activate collagen production genes

Collagen production is commonly under the control of TGF‐β signaling through SMAD proteins (Sundqvist et al., 2013). Further, RAS signaling plays a prominent role in the conversion of TGF‐β signaling from anti‐ to pro‐oncogenic and thus promotes tumor progression (Vasilaki et al., 2016). However, the mechanisms of the synergy between oncogenic RAS and TGF‐β signaling are enigmatic. It has been shown that RAS activation can switch TGF‐β family function from tumor‐suppressive to tumor‐promoting functions that will increase tumor growth and early dissemination of cancer cells (Grusch et al., 2010). It also has been shown that in cardiac myocytes extracellular acidosis can activate the RAS and ERK1/2 (Haworth, Dashnyam, & Avkiran, 2006). We hypothesized that this pathway can be hijacked by cancer cells to activate RAS through extracellular acidosis and that this will induce SMAD proteins to produce essential collagens for tumor cell survival. To test this hypothesis, first, we investigated the nuclear localization of SMAD4 in AA and NA MCF7 cells, as nuclear localization is a marker for SMAD4 transcriptional activity to see if SMAD pathway is activated in acid‐adapted cells? The nuclear expression of SMAD4 was higher in AA MCF7 cells compared to NA MCF7 while the cytoplasmic expression of SMAD4 in NA MCF7 was higher (Figure 3a,b). To study the possible role of SMAD3 in translocation of SMAD4, we investigated nuclear and cytoplasmic expression of pSMAD3 in AA MCF7 and MCF7 cells using ICC and confocal microscopy. The nuclear expression of pSMAD3 was significantly higher in AA MCF7 cells and cytoplasmic expression was lower (Figure 3c,d). We also looked at SMAD2 expression in both cells as some literatures proposed a role for it in collagen production and found no differences in nuclear or cytoplasmic expression (not shown). Then we investigated the activity of RAS pathway in acid‐adapted MCF7 and MCF10AT cells compared to their non‐adapted counterparts. We observed that expression of K‐RAS was higher in acid‐adapted cells while N‐Ras and H‐Ras were downregulated. To investigate if RAS is actually active in acid‐adapted cells, we measured the active RAS isoform using active RAS pull‐down. Active RAS was significantly higher in both acid‐adapted MCF7 and MCF10AT compared to their non‐adapted counterparts (Figure 3e). When RAS is active it will activate ERK through a signaling cascade driven by phosphorylation. pERK will phosphorylate SMAD3 through SMAD1 that eventually translocate SMAD4 to the nucleus and activate collagen production genes in cancer cells (Zong et al., 2020). To support this sequence of events, we performed Western blot and observed that ERK1/2 is phosphorylated and active in acid‐adapted cells (Figure 3e). To validate the effect of acid‐induced activation of RAS in vivo, we injected the acid‐adapted (activated RAS) and non‐adapted MCF10AT cells into flanks of SCID mice. Acid‐adapted MCF10ATtumors grew much faster and to a larger size compared to non‐adapted ones (Figure 3f). Based on our findings, we propose a role for acid‐adaptation activation of the RAS pathway to mobilize SMADs for collagen production (Figure 3f).

### 3.4 Acid‐adapted cells use collagen remodeling enzymes to engineer the niche

**Figure 4:**
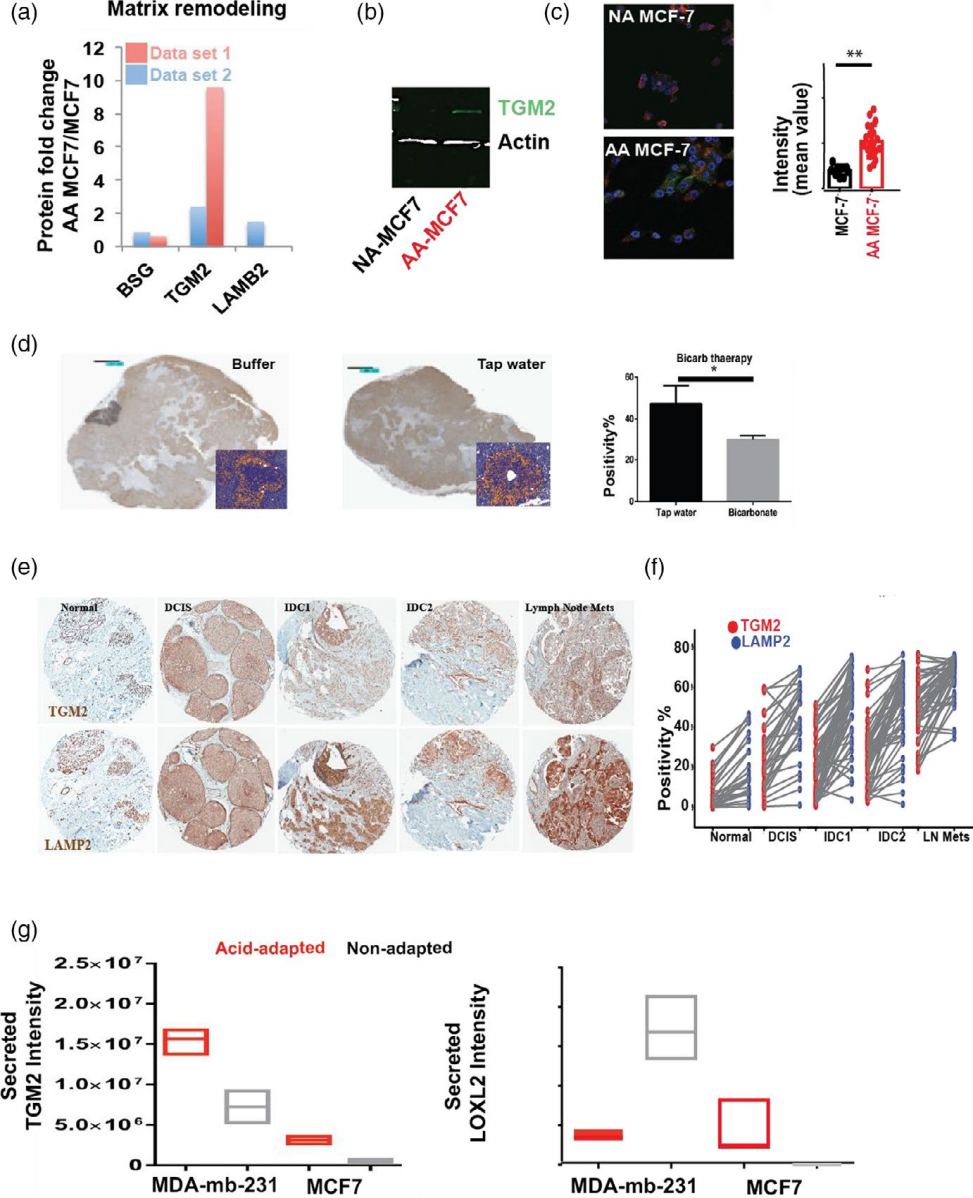
Acid‐adapted cells use collagen remodeling enzymes to engineer their niche. (a) TGM2 validation by (b) Western blot and (c) Immunocytochemistry (ICC). TGM2 expression in acid‐adapted cancer cells is significantly higher compared to non‐adapted cells. (d) Bicarbonate buffer therapy in animal reduced the expression of TGM2 compared to control group, indicating the effect of acidosis on expression of TGM2. (e, f) Translation of TGM2 expression to clinic and patient samples. LAMP2b is a marker of acidosis that we reported in our previous work. The TMA from same patients was stained for TGM2, and expression level of TGM2 and LAMP2b was compared for each sample. There is a correlation between LAMP2b and TGM2 expression in patients. (g) Acid‐adapted MCF‐7 cells not only have higher amount of TGM2 and LOXL2, but also secrete more of these enzymes to the environment. Both enzymes have been shown to play role in collagen crosslinking and stability. This strengthen our acid‐induced niche engineering phenotype of cancer cells that they build the niche they need it to survive the harsh environment such as acidosis

In our proteomic data, (Damaghi et al., 2015) we also observed that Transglutaminase 2 (TGM2) had the second highest fold change in acid‐adapted compared to non‐adapted MCF7 cells (Figure 4a). TGM2 is a calcium‐dependent enzyme of the protein‐glutamine γ‐glutamyltransferases family that makes inter‐ and/or intramolecular bonds that renders proteins resistant to proteolysis degradation. TGM2 is found both in the intracellular and the extracellular spaces of cells and plays different role in different spaces. The extracellular form binds to proteins in ECM such as collagen and fibronectin and promotes cell adhesion, ECM stabilization, wound healing, and cellular motility (Agnihotri, Kumar, & Mehta, 2013; Fesus & Piacentini, 2002; McConkey & Orrenius, 1997). Thus, TGM2 may enable the acid‐adapted cancer cells to engineer and change the ECM by even more advanced mechanisms that collagen deposition.

To validate the overexpression of TGM2 in our acid‐adapted cells, we performed Western blots and immunocytochemistry (ICC) on AA and NA MCF‐7 cancer cells (Figure 4b,c). Then, we showed in mice that we can reduce the expression of TGM2 using bicarbonate buffer consistent with our hypothesis about the acid‐induced nature of this protein (Figure 4d). To investigate this marker in breast cancer patient samples, first we analyzed the RNA expression of TGM2 in patients with different stages of breast cancer that showed significant increase in tumors compared to adjacent normal or other normal tissues (Figure S3). Then, we compared the expression of TGM2 and LAMP2b, previously proven as a marker of acidosis in solid tumors in human cancers, in each patient. The expression of TGM2 and LAMP2b were correlated in 204 different patients’ samples of different stages from adjacent normal to DICS, IDC 1, IDC 2, and macrometastasis (Figure 4e,f). We found that TGM2 expression is elevated by acid in tumors; however, as we discussed above, the localization of this enzyme is highly related to its activity. The protein crosslinking and niche engineering activity is assigned to extracellular TGM2. To determine if TGM2 is actually secreted to the extracellular milieu and its extracellular expression is increased in acid‐adapted cells we did secretome analysis of MCF‐7 and AA‐MCF7 using mass spectrometry‐based proteomics. The secretome analysis showed the higher expression of TGM2 in both AA MCF‐7 and AA MDA‐mb‐231compared to the corresponding non‐adapted cells (Figure 4g). We also observed other LOXL2, which is an enzyme that has been shown to play a role in collagen crosslinking and stability (Peng et al., 2017), was overexpressed in AA MCF7 (Figure 4g). Western blot analysis validated overexpression of LOXL2 in AAMCF7 cells (Figure S4) similar to TGM2. The MDA‐mb‐231 cells look like that only use TGM2 secretion to crosslink the collagen.

To further test our hypothesis in cancer patient samples we stained for TGM2 in whole mount breast tumors. The results showed higher amount of TGM2 in acidic regions such as center of DCIS and also co‐registered with collagen and other fibers in ECM and extracellular spaces (Figure S5). We also looked at the expression level of matrix metalloproteinases (MMPs) in our secretome data and found no overexpression of these proteins in the extracellular spaces of the cells (Figure S6a).

The above findings imply that adaptation to an acidic environment by cancer cells induces matrix remodeling, which can be viewed as a type of “niche engineering” to promote survival.

## 4 DISCUSSION

### 4.1 Collagen production and engineering are necessary for early stage cancer cells survival

Cancer is a dynamic system composed of cancer cells and their microenvironment that is always changing. Interactions among components of the cancer ecosystem shape the forces driving tumor evolution. Evolution of cancer cells in their local microenvironment is governed by their niche contents that create evolutionary selection forces. Tumor cells are able to alter the local environment or even build a new environment to promote their viability and growth in any emerging new environment. This phenomenon is known as “niche construction and engineering” and we believe it to be a key part of the cancer cells self‐defined fitness strategies (Gatenby & Brown, 1867). Each subtle change in tumor niche, from cancer or normal cells or stroma cells, can have profound effects on the tumor host interaction that will affect the tumor growth dynamics dramatically. We have recently shown that different sub‐population in tumor engineers the local ecology to favor its own growth and survival (Ibrahim‐Hashim et al., 2017).

The metabolic and niche remodeling changes in early cancers are components of a nonlinear evolutionary dynamics, wherein the changing microenvironment selects for phenotypes that, in turn, alter the microenvironment in ways that provide subsequent selection forces for subsequent phenotypes. Like organisms, cancer cells frequently modify local resource distributions, influencing both their microenvironments and the evolution of traits to improve fitness (Laland et al., 1999). In ecology, these processes are known as “niche engineering,” wherein one species will modify its habitat to maximize its own fitness, often to the detriment of other species within the same habitat. Examples in nature include ants and termites who construct their habitats that are the new source of selection of their own species (Firn & Jones, 2000; Jones et al., 1997; Lawton & Jones, 1993). The best example of niche engineering in cancer cells, and the most well‐studied one to date, is the pre‐metastatic niche in lymph nodes, which is thought to be mediated by tumor‐derived exosomes (Liu & Cao, 2016). Niche construction strategies of early cancer are relatively understudied.

In this paper, we show the reciprocal interaction of tumor cells and their microenvironment focusing on adaptive strategies of cancer cells in their harsh acidic environment. We found niche construction and engineering as an evolutionary adaptive strategy exploited by cancer cells to survive in emerging harsh environment that also lead them to evolve and develop into new stages. We see the adaptation of cancer cell to acidosis as a self‐defined fitness function. Acid‐adapted cell survival and proliferation are determined entirely by their own heritable phenotypic properties; that is, cells can develop independence from normal tissue control through either mutations or phenotypic plasticity and reversible adaptation that disrupt their response to the host signals. A self‐defined fitness function through plasticity or adaptation is similar to when tissue control signals are lost due to inflammation or infection (Gatenby & Brown, 1867). Here, we also show how adaptation to microenvironmental selection pressure such as acidosis regulates cancer cells self‐defined fitness as part of their evolutionary strategies to survive and invade, in this case to construct and engineer their favorable niche for growth and proliferation. Here, we showed that overexpressing some collagen genes and downregulation of the others can give the cancer cells the self‐defined fitness function that will help them reconstruct and engineer their niche in their own favor to survive, proliferate or even metastasize later.

**Figure 5:**
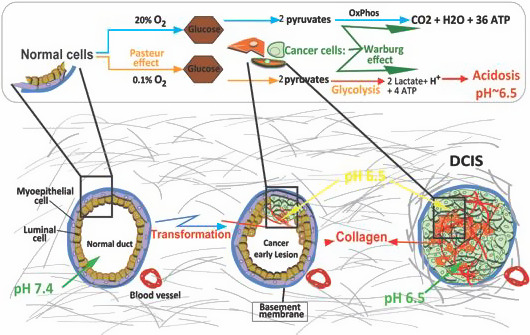
Acid‐induced collagen production promotes tumors early stage development and progress in different cancer models. Schematic of breast cancer tumors growth regarding the acidic microenvironment and collagen morphology and structure change. Solid tumors are profoundly and continuously acidic due to Pasteur and Warburg effect. The collagen morphology variation has been reported previously with mostly unknown trigger and mechanism. Here, we show how microenvironmental factors such as acidosis can promote the collagen production and its role in tumor evolution. At the early stages, acid‐adapted cancer cells inside the DCIS gain the ability of producing the extracellular matrix component such as collagen. This will enable them and probably their neighbors surviving the anoikis and progress toward next stage

In this paper, we showed for the first time the molecular biology mechanism behind the acid‐induced niche reconstruction and engineering that was also related to an evolutionary subject of self‐defined fitness. We believe evolution is theory of cancer and understanding all the evolutionary disciplines ruling the cancer growth and development will help us fighting against cancer and come up with strategies to control it. Figure 5 shows a schematic of breast cancer tumors (as representative of many epithelial solid tumors) growth regarding the acidic microenvironment and collagen morphology and structure change. Solid tumors are profoundly and continuously acidic due to Pasteur and Warburg effect. The collagen morphology variation has been reported previously with mostly unknown trigger and mechanism. Here, we show how microenvironmental factors such as acidosis can promote the collagen morphology changes over cancer progress. Acid‐adapted cells gain the ability of producing the extra cellular matrix component such as collagen that help them to survive and progress. All the above‐described changes are summarized in a phenotype: niche construction and engineering ability, and as we showed in this paper any mutation changing this phenotype can hurt the cancer cells and benefit patients.

## CONFLICT OF INTEREST

The authors declare no competing interests.

## AUTHOR CONTRIBUTIONS

M.D. conceived the study, developed the methodology, and designed and performed experiments and computational analysis. H.M., L.X., and T.C. performed the experimental work. J.J. supervised the microscopy analysis work. H.M. and A.D.B assisted with histopathology. M.D., B.F., and J.M.K. performed the proteomics and its data analysis. N. D.G. supervised the spinning disk experimental work. All authors participated in the interpretation of the results. M.D. wrote the paper with contributions from all authors. All authors have read and approved the final version of the manuscript. M.D. directed the study.

## ETHICS STATEMENT

All procedures on animals were carried out in compliance with the Guide for the Care and Use of Laboratory Animal Resources (1996), National Research Council, and approved by the Institutional Animal Care and Use Committee, University of South Florida (IACUC# R4051).

## Supporting information

Fig S1‐S6
