# Supplementary material for: Collagen production and niche engineering: A novel strategy for cancer cells to survive acidosis in DCIS and evolve
Source: Evol Appl. 2020 Nov 4;13(10):2689–703. doi: 10.1111/eva.13075 (PMC7691473; doi:10.1111/eva.13075)
Supplement: Supplementary file 1 — Fig S1‐S6 [file EVA-13-2689-s001.docx]

**Supplementary Information:**

**Figure S1.**

**
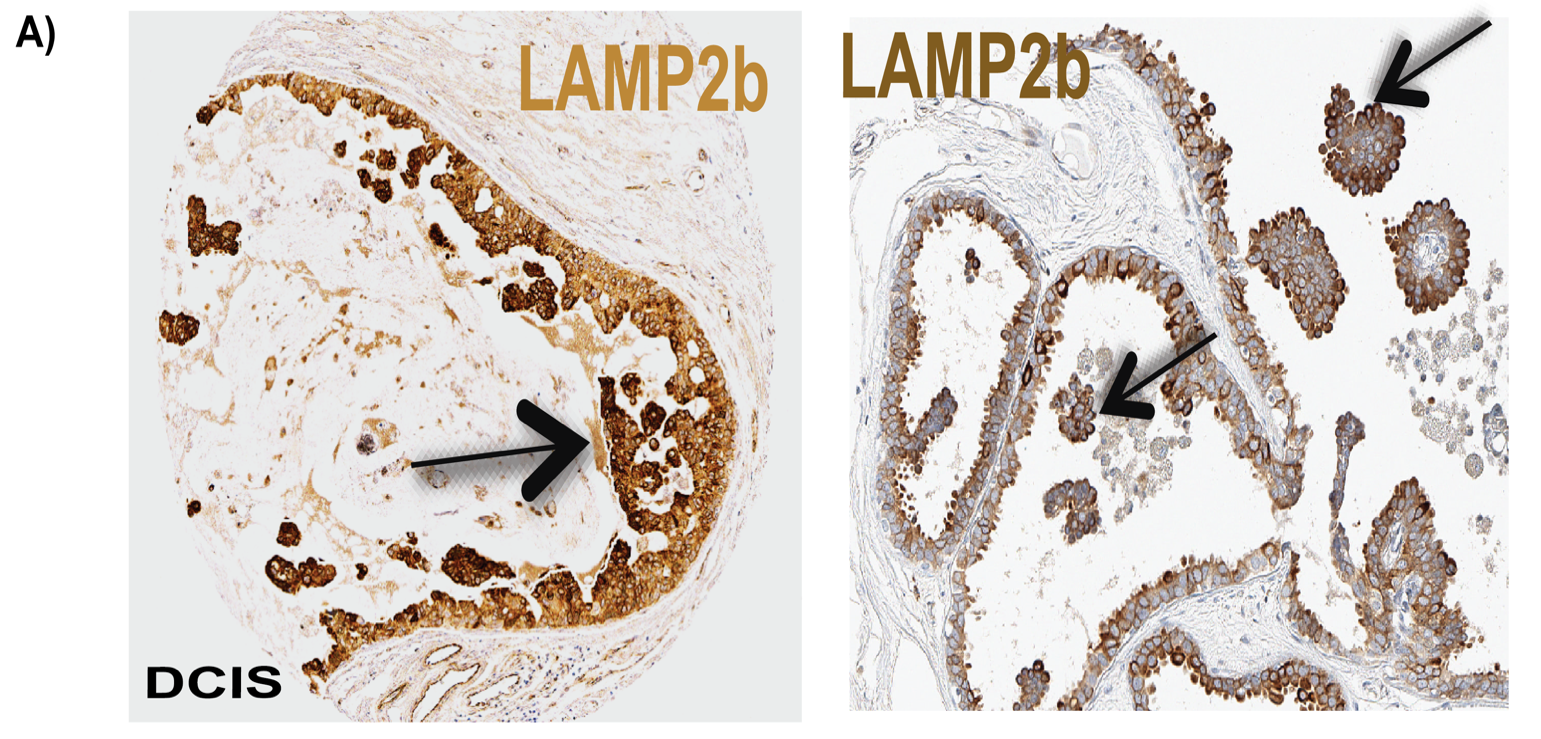
**

**Figure S1. Acid adapted cancer cells are frequently found in the center of the DCIS as one of the most acidic habitats inside the solid tumors.** The staining is LAMP2b (acid adapted phenotype marker) on two DCIS patient biopsies. The highest expression of LAMP2b (the red color with mask) is located at the center of DCIS farthest from the vasculature. There are totally separated organoid inside the duct with no ECM contact at all with high amount of LAMP2b that is a marker of acid adapted cells.

**Figure S2.**

**
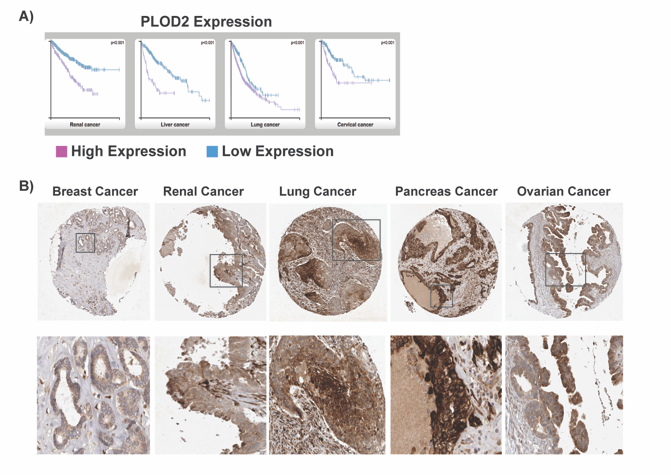
**

**Figure S2. PLOD expression in different cancer type from protein atlas.** Representative images of biopsy of different cancer types at the early stages with high expression PLOD2. Underneath each biopsy is the zoom of selected region that implies the higher expression of PLOD2 expression at the tip of growing tumors toward the inside the duct that shows the role of collagen production in cancer cells growth inside the duct. Pictures and data are extracted form human protein atlas website: www.proteinatlas.org.

**Figure S3.**


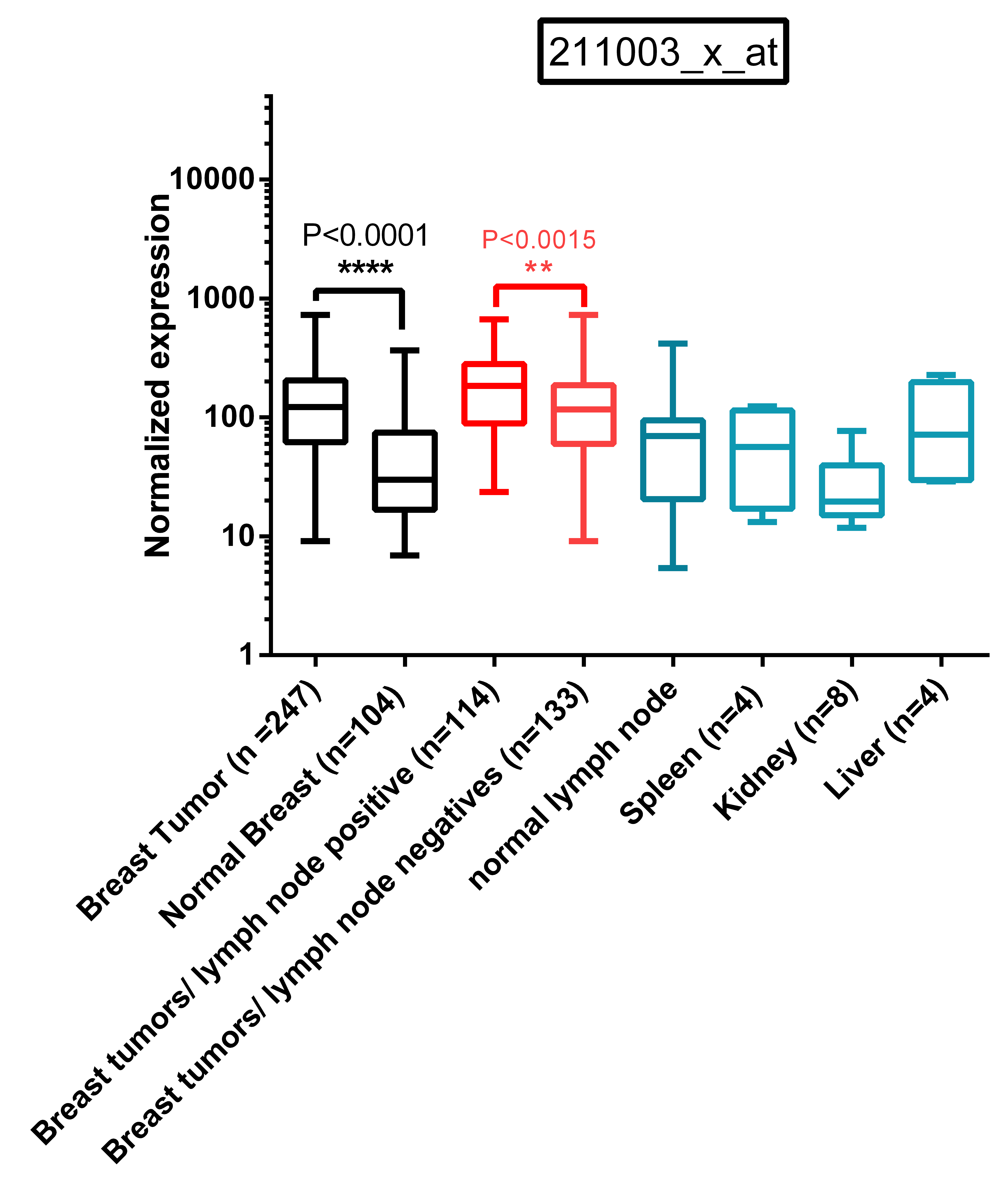


**Figure S3. Microarray analysis of Moffitt patient’s data.** Breast tumors have significantly higher amount of TGM2 compared to adjacent normal samples and other normal organs. TGM2 expression is also higher in patients with metastatic to lymph node compared to non-metastatic ones.

**Figure S4.**


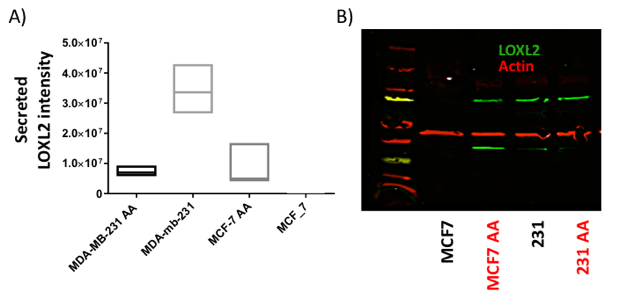


**Figure S4. LOXL2 in acid-adapted cancer cells.** Acid adapted MCF-7 cells have higher amount of LOXL2, an enzyme that has been shown play role in collagen crosslinking and stability. Western blot approved the higher expression of LOXL2 in acid adapted MCF-7 cells vs the non-adapted one. There is no change in MDA-mb-231 cells content of LOXL2. From the secretome data we assume that they use more TGM2 than LOXL2 so they didn’t need to increase the expression.

**Figure S5.
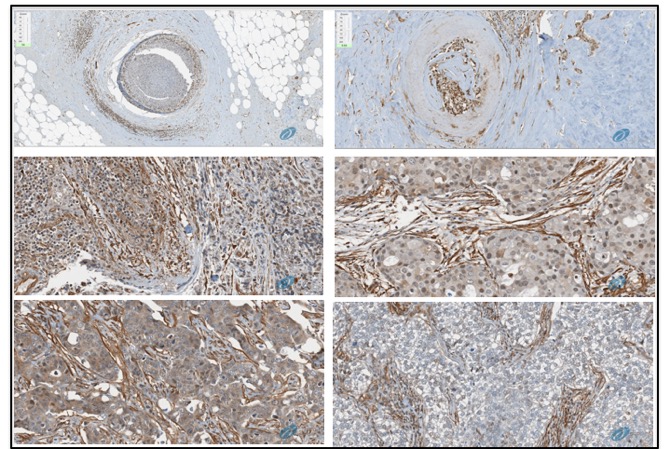
**

**Figure S5. TGM2 expression in whole mount breast tumors.** We see a lot of TGM2 protein in the acidic regions such as center of DCIS (top two images) and in the extracellular spaces and interacting with fibers confirming the crosslinking role of this protein.

**Figure S6.
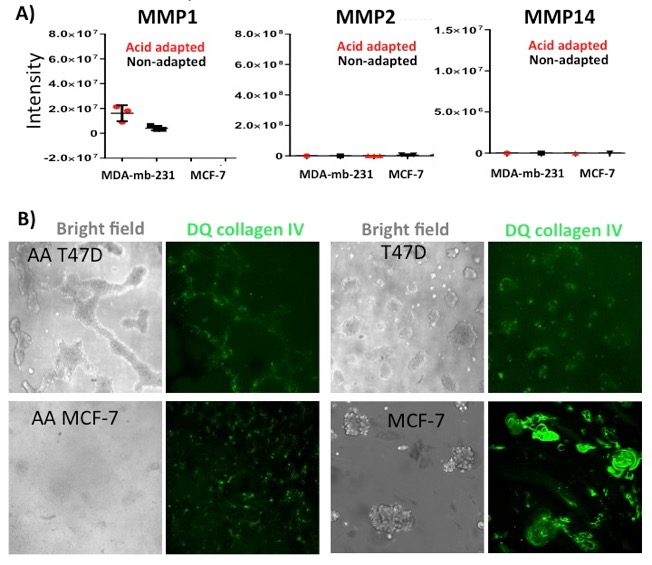
**

**Figure S6. Expression statues of MMPs in acid adapted and non-adapted cells. A)** The MMPs didn’t show any change in secretome analysis indicating that probably there is not any MMPs secreted in the conditioned media.
